# Bone-Protective Effects of Dried Plum in Postmenopausal Women: Efficacy and Possible Mechanisms

**DOI:** 10.3390/nu9050496

**Published:** 2017-05-14

**Authors:** Bahram H. Arjmandi, Sarah A. Johnson, Shirin Pourafshar, Negin Navaei, Kelli S. George, Shirin Hooshmand, Sheau C. Chai, Neda S. Akhavan

**Affiliations:** 1 Department of Nutrition, Food and Exercise Sciences, College of Human Sciences, Florida State University, Tallahassee, FL 32306, USA; sp12r@my.fsu.edu (S.P.); negin.navaei@life.edu (N.N.); ksg15c@my.fsu.edu (K.S.G.); nsa08@my.fsu.edu (N.S.A.); 2 Center for Advancing Exercise and Nutrition Research on Aging (CAENRA), College of Human Sciences, Florida State University, Tallahassee, FL 32306, USA; 3 Department of Food Science and Human Nutrition, College of Health and Human Sciences, Colorado State University, Fort Collins, CO 80523, USA; Sarah.Johnson@colostate.edu; 4 Department of Nutrition, College of Graduate and Undergraduate Studies, Life University, Marietta, GA 30060, USA; 5 School of Exercise and Nutritional Sciences, College of Health & Human Services, San Diego State University, San Diego, CA 92182, USA; shooshmand@mail.sdsu.edu; 6 Department of Behavioral Health and Nutrition, College of Health Sciences, University of Delaware, Newark, DE 19716, USA; scchai@udel.edu

**Keywords:** bioactive compounds, functional foods, menopause, nutrition, polyphenols, (poly)phenols, prune, osteopenia, osteoporosis

## Abstract

Osteoporosis is an age-related chronic disease characterized by a loss of bone mass and quality, and is associated with an increased risk of fragility fractures. Postmenopausal women are at the greatest risk of developing osteoporosis due to the cessation in ovarian hormone production, which causes accelerated bone loss. As the demographic shifts to a more aged population, a growing number of postmenopausal women will be afflicted with osteoporosis. Certain lifestyle factors, including nutrition and exercise, are known to reduce the risk of developing osteoporosis and therefore play an important role in bone health. In terms of nutrition, accumulating evidence suggests that dried plum (*Prunus domestica* L.) is potentially an efficacious intervention for preventing and reversing bone mass and structural loss in an ovariectomized rat model of osteoporosis, as well as in osteopenic postmenopausal women. Here, we provide evidence supporting the efficacy of dried plum in preventing and reversing bone loss associated with ovarian hormone deficiency in rodent models and in humans. We end with the results of a recent follow-up study demonstrating that postmenopausal women who previously consumed 100 g dried plum per day during our one-year clinical trial conducted five years earlier retained bone mineral density to a greater extent than those receiving a comparative control. Additionally, we highlight the possible mechanisms of action by which bioactive compounds in dried plum exert bone-protective effects. Overall, the findings of our studies and others strongly suggest that dried plum in its whole form is a promising and efficacious functional food therapy for preventing bone loss in postmenopausal women, with the potential for long-lasting bone-protective effects.

## 1. Introduction

The postmenopausal period typically occupies one-third of a woman’s life [1], and it is estimated that by the year 2020, more than 46 million women in the United States (U.S.) will be postmenopausal [2]. Menopause is associated with the development of numerous chronic diseases [3], due to an abrupt cessation of ovarian hormone production, namely estrogen. Osteoporosis is a chronic and debilitating age-related skeletal disease characterized by the loss of bone mass and a deterioration of the microstructural properties of bone, resulting in an increased propensity for fragility fractures [4]. Osteoporosis is responsible for more than 1.5 million fractures per year in the U.S., with most occurring in postmenopausal women [5]. Osteoporosis and its related bone fractures are a major public health concern as they are associated with increased morbidity and mortality, a poor quality of life, and a large economic burden [6,7]. Approximately 44 million men and women over the age of 50 have osteoporosis or low bone mass in the U.S. [3]. Diminished estrogen levels associated with menopause result in an initial phase of rapid bone loss, followed by a period of a slower deterioration of the skeleton [8]. This rapid phase of bone loss occurs within the first five to 10 years following the cessation of menses or the surgical removal of the ovaries [9]. As the demographic shifts to a more aged population, the prevalence and incidence of osteoporosis are likely to continue to increase, with affected individuals being at a greater risk of falls, fragility fractures, and therefore morbidity and mortality [3]. As such, therapeutic strategies that can delay, slow down, or prevent bone loss in aging individuals, and particularly in postmenopausal women, are critically needed.

Hormone replacement therapy is a logical therapeutic strategy for postmenopausal women since age-related chronic disease development typically begins after the cessation of ovarian hormone production. However, evidence from the Women’s Health Initiative indicates that the risks associated with hormone replacement therapy outweigh its benefits, making the exploration of alternative therapies necessary [10]. Although the Food and Drug Administration has approved several anti-resorptive pharmacological agents such as bisphosphonates and denosumab, as well as the bone-forming pharmacological agent teriparatide, these drugs are associated with adverse side effects, are costly, and often have low compliance [11]. Therefore, it is imperative that safe and cost-effective therapeutic strategies, aside from medications, that can delay, slow down, or prevent bone loss in postmenopausal women be identified, investigated for efficacy, and disseminated for public use.

It is well-known that certain lifestyle factors, including diet and nutrition, play an important role in bone health. In fact, research has demonstrated that certain foods (i.e., functional foods) and their bioactive compounds, including nutrient and non-nutrient compounds, have bone-protective effects [12]. Of the functional foods investigated for their bone-protective properties, the most efficacious have typically been in the form of fruits and vegetables. The findings from our studies [13,14,15] and others [16,17] suggest that dried plum (*Prunus domestica* L.) is the most effective in preventing and reversing bone loss among the fruits and vegetables investigated. This has also been demonstrated by Mühlbauer et al. [18,19], who examined the effects of 60 fruits and vegetables on bone. Their findings indicated that onion and dried plum had the most potent bone-protective effects. Though the consumption of fruits and vegetables for general health and well-being is encouraged, in terms of bone health, not all fruits and vegetables offer the same benefits. For instance, while certain fruits such as dried plum and to some extent blueberries exert bone-protective effects [20], in our observations [14], the same may not be true for other fruits such as raisins and dates. We are using a conditional term for the effectiveness of blueberry on bone, because our findings [20], as well as those of Zhang et al. [21,22], are preliminary at this point and await confirmation in human studies. The reasons for these discrepancies in the efficacy among fruits and vegetables remain unknown.

Due to the bioactive compound content and composition of dried plum, as well as promising preclinical and clinical research findings, dried plum has been and continues to be investigated as a potential functional food with respect to bone health. Numerous studies in cells, rodent models of postmenopausal osteoporosis, and postmenopausal women have demonstrated its efficacy and have identified possible mechanisms of action. Studies have aimed to identify specific bioactive compounds in dried plum responsible for its bone-protective properties. The present review provides an overview of dried plum, including its nutritional and bioactive compound composition, and provides evidence from preclinical and clinical studies supporting the efficacy of dried plum in preventing and reversing bone loss in postmenopausal women, as well as evidence to support possible mechanisms of action, and bioactive compounds in dried plum responsible for its efficacy. Additionally, gaps in the research and promising new areas of investigation are discussed.

A literature search was performed using the PubMed database and Google Scholar. The following keywords, alone and in combination, were used: bone, dried plum, menopause, osteopenia, osteoporosis, ovariectomy, ovariectomized, plum, postmenopausal, and prune. Preclinical studies using rodent models of postmenopausal osteoporosis and clinical trials with postmenopausal women were included in this review.

## 2. Dried Plum: A Promising Functional Food for Bone Health

The U.S., primarily California, is a major producer of dried plum, with approximately 99% of the U.S. and 40% of the world supply grown in California. Dried plum was previously referred to as prunes until a formal name change was requested and approved by the Food and Drug Administration in 2001 [23]. It is most commonly known for its effects on gastrointestinal motility and research has been and continues to be conducted with respect to gastrointestinal health to support these observations. In addition, dried plum has been investigated for its antimicrobial, cancer-preventive, cardiometabolic, neurological, and bone-protective effects [24,25].

To date, the most notable research has been in the area of bone metabolism and health. The unique ability of dried plum to promote bone health is likely related to its bioactive compound composition. Dried plum is rich in nutrient bioactive compounds including dietary fiber, vitamin K, boron, copper, magnesium, manganese, among others, many of which are known to positively influence bone [23,24,25]. It is also rich in non-nutrient bioactive compounds including (poly)phenols such as chlorogenic acids (e.g., chlorogenic acid, neochlorogenic acid, cryptochlorogenic acid) and proanthocyanidins [24,25]. Dried plum has been ranked as having one of the highest oxygen radical absorbance capacities among the commonly consumed fruits and vegetables. It has been suggested that this is likely to be primarily due to its (poly)phenolic compound composition and content, as dried plum is low in ascorbic acid, carotenoids, and vitamin E [23]. In addition, previous research has demonstrated that phenolic compounds, including those found in dried plum, exert bone-protective effects and profoundly affect bone metabolism [25]. For instance, rutin, a flavonoid commonly found in plums and various berries, has been reported to inhibit ovariectomy-induced bone loss in a rat model of osteoporosis [26]. Nonetheless, the question still remains as to which bioactive component(s) of dried plum are responsible for its bone-protective effects. The answer may simply be that the whole fruit is more efficacious than its isolated components due to the additive and/or synergistic effects of these components within the food matrix.

## 3. Dried Plum and Bone Health: Rodent Models of Postmenopausal Osteoporosis

**Figure 1:**
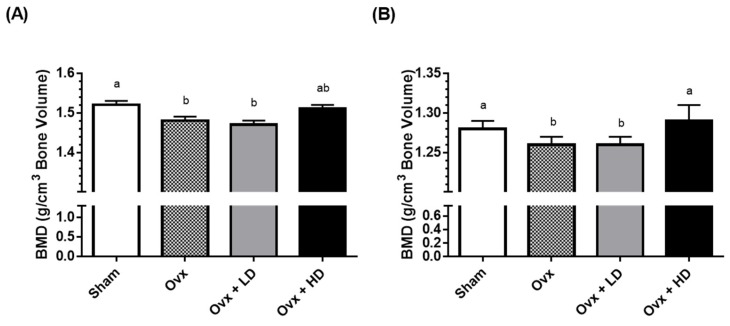
(**A**) Effects of ovariectomy and dried plum on bone density of right femur; (**B**) Effects of ovariectomy and dried plum on bone density of 4th lumbar spine. Bars represent mean ± standard error of the mean. Bars that do not share the same letters are significantly (*p* < 0.05) different from each other. BMD, bone mineral density; HD, high dose (25%) dried plum; LD, low dose (5%) dried plum; Ovx, ovariectomized; Sham, sham-operated.

Previous studies investigating the role of dried plum on bone health in rodent models of postmenopausal osteoporosis are summarized in Table 1. Our laboratory [27] was the first to report the bone-protective effects of dried plum both in general, and specifically in an established rat model of postmenopausal osteoporosis [28]. We showed that ovariectomy led to significant declines in the bone mineral density (BMD) of the 4th lumbar vertebrae and femurs, as well as a decrease in the tibial trabecular bone area compared to sham-operated (Sham) rats. However, ovariectomized (Ovx) rats that received 25% of the diet as dried plum for 45 days did not lose bone, while those receiving 5% of the diet as dried plum lost bone similarly to Ovx rats not receiving dried plum (Figure 1). Despite this, we also found that dried plum dose-dependently increased circulating insulin-like growth factor-I (IGF-I) levels, while having no effect on tartrate-resistant acid phosphatase-5b (TRAP-5b) levels. At that time, TRAP-5b was considered to be a biomarker of bone resorption. Therefore, these results suggested that dried plum prevented bone loss, in part, by increasing the rate of bone formation, but not through inhibiting bone resorption.

**Figure 2:**
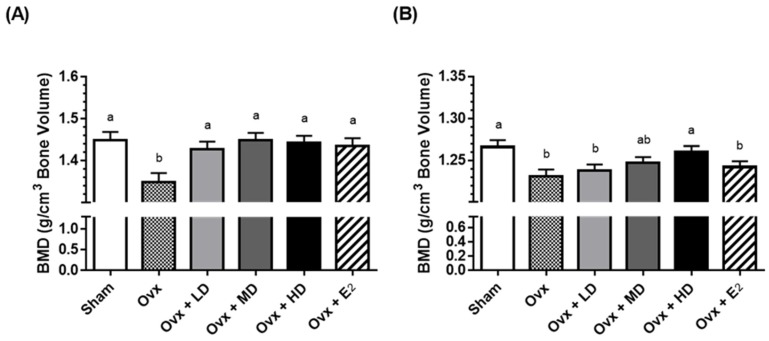
(**A**) Effects of ovariectomy, dried plum, and estrogen on bone density of right femur; (**B**) Effects ovariectomy, dried plum, and estrogen on bone density of 4th lumbar spine. Bars represent mean ± standard error of the mean. Bars that do not share the same letters are significantly (*p* < 0.05) different from each other. BMD, bone mineral density; E_2_, 17β-estradiol, LD, low dose (5%) dried plum; high dose (25%) dried plum; MD, medium dose (15%) dried plum; Ovx, ovariectomized; Sham, sham-operated.

**Figure 3:**
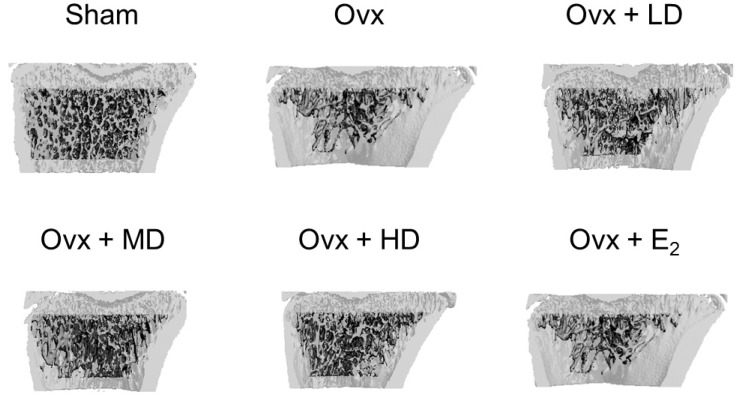
Representative images of proximal tibia demonstrating the effect of ovariectomy, dried plum, and estrogen on trabecular bone structure. E_2_, 17β-estradiol; LD, high dose (25%) dried plum; low dose (5%) dried plum; MD, medium dose (15%) dried plum, Ovx, ovariectomized; Sham, sham-operated.

We next asked the question: could the addition of dried plum to the diet restore bone mass after bone loss has occurred? Toward this end, our laboratory [27] used an Ovx rat model of postmenopausal osteoporosis to evaluate the ability of dried plum at different doses to reverse bone loss. In this study, 90-day-old female Sprague-Dawley rats were divided into six groups: Sham control, Ovx control, Ovx + 17β-estradiol (E_2_), Ovx + 5% of the diet as dried plum, Ovx + 15% of the diet as dried plum, and Ovx + 25% of the diet as dried plum for 60 days. Interestingly, the consumption of all doses of dried plum (5%, 15%, and 25%) effectively restored femoral and tibial BMD to the same extent as E_2_, while only the group receiving 25% dried plum showed the restoration of 4th lumbar BMD (Figure 2). At the time in which this study was conducted, the loss of bone volume accompanied by the loss of trabecular connectivity was generally believed to be an irreversible process [29]. To our knowledge, our study was the first to demonstrate that an agent of any kind could reverse the loss of trabecular microstructures including the bone volume/total volume, connectivity density, trabecular number, trabecular separation, and structure model index (Figure 3).

We next sought to evaluate the efficacy of several potentially bone-protective functional foods and bioactive compounds (i.e., dried plum, figs, dates, raisins, blueberries, dried plum (poly)phenols, fructooligosaccharides (FOS), and β-hydroxy-β-methylbutyrate, alone and in combination, on the bone mass and quality in Ovx rats after bone loss had already occurred [14]. Our findings revealed that the group receiving 5% FOS + 7.5% dried plum in their diet had the greatest reversal of BMD loss in the right femur and 4th lumbar spine, 4th lumbar spine calcium loss, and trabecular separation. Interestingly, none of the treatments altered the serum or urinary markers of bone turnover. The findings of this study suggest that the addition of prebiotic FOS to dried plum improves its efficacy in restoring bone mass and quality after bone loss has occurred. Although the mechanisms remain unknown, FOS has been demonstrated to increase the absorption of minerals, including calcium and magnesium, in the colon [30]. Additionally, considering that FOS is a prebiotic, it is likely that the combination of FOS and dried plum worked through gut microbiota-related mechanisms including an increase in mineral absorption. Importantly, the dose of dried plum (i.e., 7.5% of the diet) that was efficacious in combination with FOS was lower than what was previously found to be effective. This suggests that the addition of FOS to dried plum may improve the efficacy of dried plum and should be further investigated. These findings will be particularly important if they can be translated to humans, as it could be a novel strategy for reducing the amount of dried plum an individual would need to consume on a daily basis to achieve the same outcomes.

In addition to demonstrating the bone-protective effects of dried plum alone and in combination with FOS, we have also demonstrated in numerous studies that soy is a potentially efficacious functional food for promoting bone health [31,32,33,34,35]. We therefore sought to determine the efficacy of a soy-based diet in combination with 7.5% dried plum, 5% FOS, or 7.5% dried plum + 5% FOS on reversing bone loss in a rat model of postmenopausal osteoporosis. We found that the combination of a soy-based diet with dried plum, FOS, or both, significantly improved the whole body BMD and femoral BMD, while the combination of all components (i.e., soy, dried plum, and FOS) had the most pronounced effect on lumbar BMD [15]. All interventions were noted to improve the biomechanical properties of bone, as demonstrated by the increased ultimate load. The serum biomarker results suggest that these improvements may have been due, in part, to their ability to enhance bone formation and reduce bone resorption, as shown by increases in blood alkaline phosphatase (ALP) and decreased urinary deoxypyridinoline (Dpd).

Other investigators have also demonstrated the bone-protective effects of dried plum in rodent models postmenopausal osteoporosis. Rendina et al. [16] showed that feeding 25% of the diet as dried plum for four weeks to female Ovx C57BL/6J mice prevented the loss of BMD and bone mineral content (BMC) of the spine, and trabecular microarchitectural properties of the vertebrae and proximal tibiae, resulting in greater bone strength and stiffness in the vertebrae. Additionally, feeding dried plum in the diet at 15% and 25% doses restored myeloid and lymphoid levels to that of the sham-operated mice and suppressed ex vivo concanavalin A stimulated lymphocyte tumor necrosis factor-α production in splenocytes. 

In another study, Rendina et al. [36] reported the results of a study comparing the bone-protective effects of dried plum with other fruits (i.e., dried apple, apricot, grape, and mango) in Ovx C57BL/6 mice over an eight-week period. They demonstrated that dried plum had superior anabolic effects on the trabecular bone microarchitectural properties of the vertebrae, was able to prevent tibial bone loss, and restored the trabecular biomechanical properties of the spine when compared with the other fruits. In addition, dried plum was more efficacious than the other fruits in enhancing plasma glutathione peroxidase activity, downregulating osteoclast differentiation, upregulating osteoblast activity, and suppressing Ovx-induced apoptosis. 

Smith et al. [37] reported the findings of a study out of the same laboratory in which they compared the effects of six weeks of dried plum supplementation to treatment with parathyroid hormone (PTH). Treatment with dried plum at doses of 15% and 25% in the diet restored the whole body and femoral BMD to that of the Sham group and improved the trabecular bone volume and cortical thickness. Systemic blood biomarkers of bone metabolism (i.e., *N*-terminal procollagen type 1 and Dpd) were reduced, indicating a reduction in bone turnover. Dynamic bone histomorphometric analysis of the tibial metaphysis revealed that dried plum restored the Ovx-induced increase in the cancellous bone formation rate and mineralizing surface to that of the Sham group. Dried plum also upregulated the gene expression of *bone morphogenetic protein 2*, a regulator of osteogenesis, and *IGF-I* while downregulating the *nuclear factor T cell activator 1*, a transcription factor involved in the regulation of osteoclast differentiation. Compared with that of the effects of PTH on bone, dried plum reduced the rate of bone turnover, rather than increasing the rate of bone formation. 

In summary, our data and others strongly suggest that dried plum is an efficacious functional food for preventing and reversing the loss of bone mass and structural properties in a rat model of postmenopausal osteoporosis. We have also shown that dried plum dose-dependently increases systemic and local indices of bone formation, e.g., serum and mRNA levels of ALP. These findings suggest that dried plum prevents and reverses bone loss, primarily through enhanced bone formation.

## 4. Dried Plum and Bone Health: Clinical Trials in Postmenopausal Women 

Previously conducted clinical trials investigating the role of dried plum on bone health in postmenopausal women are summarized in Table 2. To our knowledge, our laboratory was the first to evaluate the bone-protective properties of dried plum in humans. Initially, we reported the findings of a three-month clinical trial evaluating the efficacy of dried plum versus dried apple (comparative control) on the biomarkers of bone formation in postmenopausal women [41]. Here, we showed that the consumption of 100 g/day dried plum significantly increased the serum markers of bone formation, namely total ALP, bone-specific ALP (BALP), and IGF-1 by 12, 6, and 17%, respectively. The increase in BALP is important as studies have shown that clinically relevant doses of bone-forming agents such as sodium fluoride, growth hormone, and PTH take several months to moderately increase the serum levels of BALP [42]. Interestingly, the serum and urinary biomarkers of bone resorption were not affected by either intervention. These observations further support our hypothesis that dried plum prevents and reverses bone loss through enhanced bone formation.

**Figure 4:**
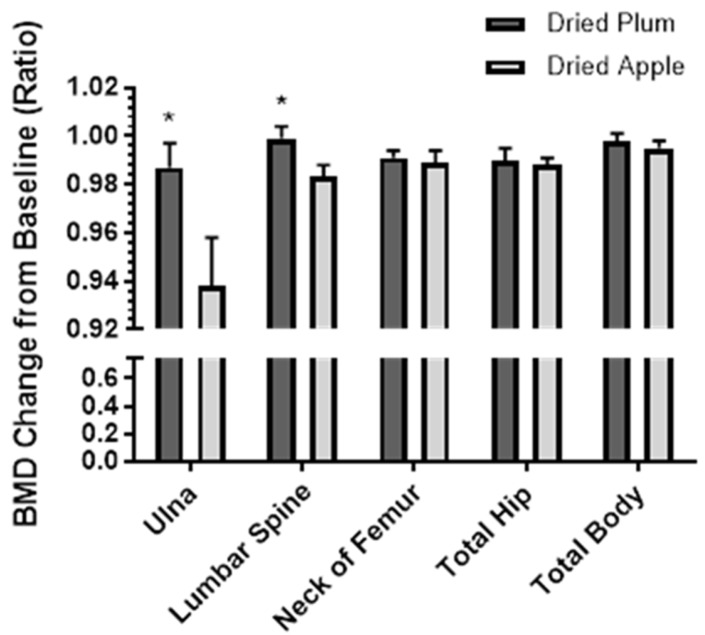
Change from baseline (ratio) in bone mineral density (BMD) from baseline to one-year following daily consumption of 100 g dried plum or 75 g dried apple. Bars represent mean ± standard error of the mean. * Denotes significant (*p* < 0.05) difference between groups.

To evaluate whether a longer treatment period would lead to an improvement in bone mass, we conducted a one-year clinical trial comparing the effects of the daily consumption of 100 g dried plum to 75 g dried apple (comparative control) on BMD and the biomarkers of bone turnover in 100 osteopenic postmenopausal women. We found that dried plum consumption significantly improved the BMD of the ulna and lumbar spine compared with the dried apple control (Figure 4) [13]. Additionally, dried plum consumption led to significantly decreased serum TRAP-5b and BALP levels. Osteocalcin and C-reactive protein levels were significantly lower in the dried plum group than in the apple group. Although the findings of our initial three-month clinical trial indicated that BALP levels increased following dried plum consumption, this was not observed in our one-year clinical trial. We cannot offer a definitive reason for this discrepancy. However, variation is inherent in bone biomarkers due to intra- and inter-individual variability, analytical reasons, and sample stability. For these reasons, BMD remains the gold-standard for evaluating bone health [43].

We later investigated the potential mechanisms of action by which dried plum exerted these effects. We showed that compared to the baseline, dried plum increased receptor activator of nuclear factor kappa-B ligand levels by +1.99% versus +18.33% in the control group, increased the osteoprotegerin levels by +4.87% versus −2.15% in the control group, and decreased serum sclerostin levels by −1.12% in the dried plum group versus +3.78% in the control group [44]. While these percent changes did not reach statistical significance, they are clearly promising preliminary findings. Collectively, these findings suggest that dried plum prevents bone loss in postmenopausal women through the suppression of bone turnover.

Next, we [45] reported the findings of a six-month clinical trial evaluating the effects of resistance training with and without dried plum at a dose of 90 g in postmenopausal breast cancer survivors. While both groups were found to increase upper and lower body strength, no improvements were observed in the body composition or BMD. Interestingly, both groups displayed improvements in the blood biomarkers of bone turnover, with no added effect of dried plum observed.

Our successive six-month clinical trial evaluated the efficacy of two doses of dried plum (50 g versus 100 g) in preventing bone loss in older postmenopausal women [46]. Our findings confirmed dried plums’ ability to prevent the loss of total body BMD and indicated that a lower dose of dried plum (i.e., 50 g) may be as effective as 100 g of dried plum. This study also demonstrated a reduction in serum TRAP-5b in both dried plum groups. 

**Figure 5:**
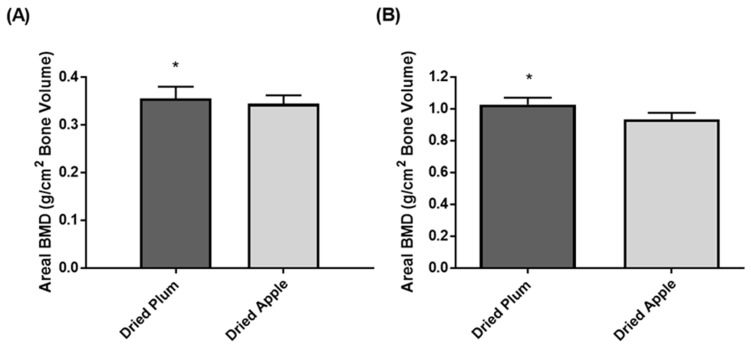
Bone mineral density (BMD) of the (**A**) ulna and (**B**) lumbar spine five years following one-year intervention study with daily consumption of 100 g dried plum or 75 g dried apple. Bars represent mean ± standard error of the mean. * Values were significantly (*p* < 0.05) different between groups.

Most recently, we evaluated whether or not individuals who received the dried plum intervention in our previous one-year clinical trial conducted five years prior were able to retain BMD to a greater extent than those who received the dried apple intervention. Of the 100 women who completed the initial clinical trial, 20 came back for a follow-up visit. All participants, irrespective of group assignment, reported that they did not regularly consume dried plums. We found that individuals that received the dried plum intervention (*n* = 8) in our previous one-year clinical trial retained BMD of the ulna and lumbar spine to a greater extent than those who received the dried apple intervention (*n* = 12) (unpublished data, Figure 5). Although these findings are preliminary, they suggest that women in the dried plum group retained bone density in the lumbar spine and ulna to a greater extent than those in the dried apple group over the course of five years, even in the absence of regular dried plum consumption. These findings should be interpreted with caution as the influence of other factors including diet, physical activity, and medications have not been evaluated. Nonetheless, these findings are promising and further research is needed to evaluate the extent to which bone density is retained following the cessation of an intervention with dried plum.

## 5. Bioactive Compounds and Possible Mechanisms of Action

Dried plum is known to contain several bioactive compounds including dietary fiber, vitamins (e.g., vitamin K), minerals (e.g., boron, copper), and (poly)phenolic compounds such as chlorogenic acids (i.e., chlorogenic acid, neochlorogenic acid, and cryptochlorogenic acid) and proanthocyanidins [24,25]. The exact nutrients and/or components contributing to the bone-protective effects of dried plum are unknown. However, many of these compounds are known to exert bone-protective effects and therefore likely work additively and/or synergistically.

Among the compounds found in dried plum is boron, which is a trace element critical for bone health as a deficiency or excess in consumption of boron can be harmful to bone. Dried plum contains a higher amount of boron than most fruits. In fact, the content of boron in 47.7 g dried plum (~5 dried plums) is about 1.1 mg [23]. The average daily intake of boron is about 1–2 mg/day, depending on sex and age, among other factors [47]. Boron has been shown to stimulate bone growth and bone metabolism [48], and play an important role in preserving BMD, bone microarchitecture, and bone strength [49,50,51]. A study by Chapin et al. [52] showed that rats fed diets containing 20 or more mg boron/100 g diet had a significantly improved vertebral strength. These authors also demonstrated that rats fed a boric acid diet of 200 ppm or more for nine weeks not only had a significant increase in the boron content of their bone, but retained their bone boron content three-fold greater than the controls 32 weeks after exposure to the diet ended. This suggests that boron has the ability to accumulate in bone, and though the half-life of boron in bone is uncertain, it may be similar to that of calcium, where the half-life is significantly longer than in tissue. This action of boron may play a role in the maintenance of bone density over long periods of time, even without regular consumption.

Another mineral abundant in dried plum important for bone health is potassium [53,54]. One hundred grams of dried plum contains 732 mg. Tucker et al. [55] investigated both the cross-sectional and longitudinal relationships between potassium and BMD using a Framingham Heart Study database and concluded that potassium contributes to the maintenance of BMD in men and women. Additionally, higher intakes of potassium have been shown to reduce bone resorption, particularly in the face of high protein intake [56]. 

Dried plum is also a good source of copper. A 47.7 g serving of dried plum provides 0.13 mg, which is 31.2% of the dietary reference intake. Copper is a cofactor for lysyl oxidase, which is involved in the cross-linking of collagen and elastin [24,57]. In an in vivo study by Yee et al. [57], Ovx Sprague-Dawley rats placed on a copper deficient diet had more severe osteopenia than the copper adequate diet. Copper may in part contribute to the beneficial properties of dried plum on bone, giving an additive effect. 

Dried plum is rich in vitamin K and a 47.7 g serving of dried plum provides 348 mg vitamin K, or 15.6% of the dietary reference intake [53]. Vitamin K is important for bone health as it promotes a calcium balance [58]. Additionally, it is a cofactor for the γ-carboxylation of osteocalcin, a bone matrix protein secreted by osteoblasts that promotes normal bone mineralization by regulating the growth of hydroxyapatite crystals [58]. A study by Braam et al. [59] demonstrated that supplementation with vitamin K at the level of 1 mg daily for three years attenuated the loss of BMD in the lumbar spine. 

Among the (poly)phenols found in dried plum, chlorogenic acids (e.g., neochlorogenic acid, cryptochlorigenic acid, and chlorogenic acid) are the most abundant. In fact, 100 g of dried plum is estimated to contain between 108 and 153 mg chlorogenic acids. Previous research has demonstrated that chlorogenic acids are bone-protective. For instance, Zhou et al. [60] demonstrated that supplementing Ovx rats with chlorogenic acid led to improved BMD and microarchitecture, and an increased proliferation of osteoblast precursors and osteoblast differentiation, as well as increases in bone formation biomarkers. Despite this, Léotoing et al. [40] demonstrated that the bone-protective effects of dried plum is not dependent on the content of chlorogenic acids. As the authors pointed out, it is important to consider that dried plum contains other (poly)phenols such as quercetin, rutin, proanthocyanidins, among others, as well as dietary fiber in the form of soluble and insoluble fiber including pectin, fructans, hemicelluloses, and cellulose [23,40], which may be responsible for the effects of dried plum on bone. (Poly)phenols and their metabolites are known to not only act as antioxidants themselves [53,61,62], but to also activate endogenous antioxidant and inhibit inflammatory signaling pathways [63,64]. Considering that bone loss has been linked to oxygen-derived free radicals in the bone microenvironment, an imbalance in antioxidant defenses, and oxidative stress [65,66], as well as a pro-inflammatory state [67], it is possible that other dried plum (poly)phenols and their metabolites work through this mechanism. In fact, we and others have demonstrated the antioxidant and anti-inflammatory properties of dried plum [68,69]. Dried plum has a high oxygen radical absorbance capacity (ORAC) compared with many commonly consumed fruits and vegetables [70]. More recently, Kayano et al. [61], have isolated several ortho-diphenolic and mono hydroxyl phenolic compounds with ORAC values as high as 4.68 units of Trolox equivalent per mg of dried plum. Therefore, the beneficial effects of dried plum on bone may be partly mediated through its antioxidant properties.

Importantly, dried plum is rich in soluble and insoluble fibers, including pectin, fructans, hemicelluloses, and cellulose [23,40], which are known to increase mineral absorption (e.g., calcium) [71]. For instance, fibers are fermented by colonic bacteria, resulting in the production of short-chain fatty acids (SCFA). SCFAs enhance calcium absorption through reductions in the pH of the intestinal lumen and increasing the solubility of calcium, thereby facilitating passive diffusion through exchanges of luminal calcium with cellular hydrogen, and increasing the permeability of gut epithelial cells and paracellular transport [40,71,72]. Dietary fiber, including prebiotic dietary fibers, as well as (poly)phenolic compounds, have been shown to alter the microbial composition in the gut [71,73,74]. As such, it is possible that chronic dried plum consumption induces changes in the gut microbiome, thereby increasing SCFA production. It is also possible that these changes in the gut microbiome promote bone health through other mechanisms including those involving the immune system. However, this has yet to be investigated and is therefore speculative.

Lastly, previous research has shown that dried plum rich in chlorogenic acid increases bone calcium retention in an Ovx rodent model of postmenopausal osteoporosis [39]. Although changes in biomarkers of bone turnover were not noted, the observation that bone calcium retention was greater is consistent with the notion that dried plum reduces bone turnover. This is an interesting finding that warrants further investigation.

## 6. Conclusions

The bone-protective effects of dried plum in postmenopausal women have been supported by several animal studies and confirmed in randomized controlled trials. The exact mechanisms by which dried plum exerts these effects remains unknown. Additionally, it is unclear as to which bioactive compound(s) in dried plum is/are responsible for its bone-protective properties. Considering that many of the bioactive compounds present in dried plum have been shown to modulate bone metabolism, it is likely that there are additive and/or synergistic effects among these compounds. In addition to preventing bone loss in postmenopausal women, our recent findings suggest that the bone-protective effects of dried plum may be long-lasting, thereby contributing to the maintenance of bone density after regular consumption has ceased.

The findings of our previously conducted clinical trials indicate that dried plum is most efficacious in preventing bone loss in the ulna and lumbar spine. Although the reason for this is not currently known, both vertebra and ulna contain more trabecular bone than other sites (e.g., femur). In fact, vertebra and ulna contain more than 60% and up to 50% trabecular bone, respectively. Bone turnover is known to be greater in trabecular bone than cortical bone [75,76]. The body of literature suggests that dried plum slows the rate of bone turnover. As such, it is likely that dried plum has a more pronounced effect of reducing bone loss at these sites through reductions in trabecular bone turnover.

An important point to consider with any nutritional intervention, including functional foods, is caloric intake. The addition of dried plum to the diet contributes approximately 120 kcal and 240 kcal for 50 g and 100 g doses, respectively [23]. In our previous clinical trials, study participants were advised to maintain their usual diet and physical activity patterns throughout the duration of the study. We did not observe significant changes in nutritional intake, including calories, nor in body weight. Considering that self-reported dietary intake and physical activity are known to have limitations, including measurement error, it is therefore unknown as to whether our study participants made alterations in their diets or physical activity to maintain their body weight. Dried plum is high in dietary fiber which alters the transit time and may increase satiety, thereby reducing the overall caloric intake. Logically, one would assume that the addition of dried plum to the diet would displace other calories. However, this is speculative and needs future confirmation. 

Future research should aim not only to better understand the mechanisms by which dried plum consumption contributes to bone health, including the involvement of the gut microbiome, but also to elucidate the long-term maintenance of such effects, including their role in fracture prevention. With respect to feasibility, we have observed good study participant treatment compliance and retention with long-term dried plum consumption at doses of 50 g (six months) and 100 g (one year). Study participants were generally willing to commit to the regular consumption of dried plum in order to improve their bone health and avoid taking osteoporosis medications. Our study participants were educated on ways to incorporate dried plum into their diets (e.g., in their meals) in an effort to promote compliance. Nonetheless, there are challenges with any long-term dietary intervention. For this reason, it is important that future studies aim to establish the lowest dose of dried plum or alternative dosing methods (e.g., intermittent dosing regimens) that provide the same efficacy as 100 g/day. Additionally, the combination of other foods and/or bioactive compounds that show promise for bone health, such as FOS, with dried plum should be further investigated, as this may enhance its efficacy. Finally, many of the previously conducted clinical trials evaluating the bone-protective effects of dried plum had small sample sizes, which may have contributed to null findings. Therefore, future large-scale clinical trials are needed to further establish the bone-protective effects of dried plum in postmenopausal women.

## Figures and Tables

**Table 1:** Preclinical studies investigating the role of dried plum on bone health in rodent models of postmenopausal osteoporosis.

Reference	Model	Number	Groups	Duration	Primary Outcomes	Primary Findings
Arjmandi et al., 2001 [27]	Sham and Ovx female 90-day old Sprague-Dawley rats	48	(1) Sham control, (2) Ovx control, (3) Ovx + 5% dried plum, or (4) Ovx + 25% dried plum	45 days	BMD and % mineral content of right femur and 4th lumbar vertebrae; trabecular total area and bone area; cortical total area, bone area, marrow space, endosteal perimeter, and periosteal perimeter; serum ALP, TRAP, and IGF-1	Compared to Ovx control: 4th lumbar vertebrae and femur BMD and trabecular bone area were significantly ↑ in the 25% dried plum group
Deyhim et al., 2005 [38]	Sham and Ovx female 90-day old Sprague-Dawley rats	80	(1) Sham control, (2) Ovx control, (3) Ovx + 17β-estradiol, (4) Ovx + 5% dried plum, (5) Ovx + 15% dried plum, (6) Ovx + 25% dried plum	60 days	BMD of femur, left tibiae, and 4th lumbar; biomechanical properties (length, cortical area, unit yield force, and unit ultimate force); trabecular microarchitectural properties (BV/TV), Tb N, Tb S, Tb Th, ConnDens, SMI); serum IGF-1, ALP, and TRAP; urinary Dpd	Compared to Ovx control: Femur and tibia BMD were ↑ in all dried plum groups, 4th lumbar BMD tended to be significantly and was significantly ↑ in the 15% and 25% dried plum groups, respectively; unit yield force tended to be significantly ↑ in all dried plum groups; Tb S was significantly ↑ in all dried plum groups while Tb N and Tb Th were significantly ↑ in 15% and 25% dried plum groups and BV/TV and ConnDens were significantly ↑ in 25% dried plum group; urinary Dpd tended to be significantly ↓ in all dried plum groups
Arjmandi et al., 2010 [14]	Sham and Ovx female 90-day old Sprague-Dawley rats	180	(1) Sham control, (2) Ovx control, (3) Ovx + 2% FOS, (4) Ovx + 5% FOS + 7.5% dried plum, (5) Ovx + 2% FOS + 5% dried plum, (6) Ovx + 2% FOS + dried plum polyphenols, (7) Ovx + 2% FOS + dried plum juice, (8) Ovx + 2% FOS + dried plum puree, or (9) Ovx + 2% FOS + dried plum pulp/skins	60 days	BMD and BMC of whole body, right femur, and 4th lumbar vertebrae; bone histomorphometric parameters (BV/TV, Tb N, Tb S, Tb Th, ConnDens, and SMI); 4th lumbar calcium content; serum osteocalcin and IGF-1; serum and urinary calcium, magnesium, and phosphorus; urinary Dpd	Compared to Ovx control: 4th lumbar vertebrae and femur BMD were significantly ↑ in 5% FOS + 7.5% dried plum group with tendency to significantly ↓ loss of lumbar vertebrae calcium, and significantly ↓ trabecular separation
Johnson et al., 2011 [15]	Sham and Ovx female 90-day old Sprague-Dawley rats	72	(1) Sham control, (2) Ovx control, (3) Ovx + soy, (4) Ovx + soy + dried plum, Ovx + soy + FOS, or (5) Ovx + soy + dried plum + FOS	60 days	BMD and BMC of the whole body, right femur, and 4th lumbar vertebrae; femoral strength; bone histomorphometric parameters (BV/TV, Tb N, Tb S, Tb Th, and MS/BS); serum total ALP; urinary creatinine and Dpd	Compared to Ovx control: Whole body and 4th lumbar BMD tended to be significantly ↑ in Ovx + soy + dried plum group and was significantly increased in Ovx + soy + dried plum + FOS group, and right femur BMD was significantly ↑; Tb Th tended to be significantly ↑ and Tb Sp, and MS/BS tended to be significantly ↓ in Ovx + soy + dried plum + FOS group; serum ALP and urinary Dpd tended to be significantly ↓ in Ovx + soy + dried plum group and urinary Dpd was significantly ↓ in Ovx + soy + dried plum + FOS group
Rendina et al., 2012 [16]	Sham and Ovx female 90-day old C57BL/6J mice	59	(1) Sham control, (2) Ovx control, (3) Ovx + 5% dried plum, (4) Ovx + 15% dried plum, (5) Ovx + 25% dried plum	4 weeks	BMA, BMC, and BMD of the 4th to 5th lumbar vertebrae; bone microarchitecture parameters of tibia and 4th lumbar vertebrae (BV/TV, Tb N, Tb S, Tb Th, ConnDens, and SMI); biomechanical properties of trabecular bone (total force, stiffness, size-independent stiffness, and Von Mises stresses); plasma PINP and IGF-1; Runx2, osteocalcin, and NFATc1 bone gene expression	Compared to Ovx control: BMC and BMD were significantly ↑ in 25% dried plum group; BV/TV and vertebra Tb N were significantly ↑ in 15% and 25% dried plum groups, tibia Tb N was increased in 25% dried plum group, tibia Tb S was significantly ↓ in 15% and 25% dried plum groups while vertebra Tb S was significantly decreased in 25% dried plum group; vertebra ConnDens and SMI were significantly ↑ and ↓, respectively, in 15% and 25% dried plum groups while vertebra apparent mean/density tended to be ↑ in 15% dried plum group and was significantly ↑ in 25% dried plum group; tibia ConnDens and SMI, were significantly ↑ and ↓, respectively, in 15% and 25% dried plum groups while apparent mean density was increased in 25% dried plum group; vertebra total force, stiffness, and size-independent stiffness were significantly ↑ and Von Mises stresses was significantly ↓ in 15% and 25% dried plum groups; PINP, NFATc1, and Runx2 were significantly ↓ in all dried plum groups while IGF-1 was significantly ↑ in 15% dried plum group and osteocalcin was significantly decreased in 15% and 25% dried plum groups
Rendina et al., 2013 [36]	Sham and Ovx female 90-day old C57BL/6J mice	68	(1) Sham control, (2) Ovx control, (3) Ovx + 25% dried plum, (4) Ovx + 25% dried apple, (5) Ovx + 25% dried apricot, (6) Ovx + 25% dried grape, (7) Ovx + 25% dried mango	8 weeks	BMA, BMC, and BMD of the whole body and 4th and 5th lumbar vertebrae; bone microarchitecture parameters of tibia and 4th lumbar vertebrae (BV/TV, Tb N, Tb S, Tb Th, ConnDens, SMI, and trabecular density); biomechanical properties of trabecular bone (total force, stiffness, size-independent stiffness); plasma GPx; bone marrow gene expression of NFATc1, Col1a1, ALP, osteocalcin, Bak1; flushed femur expression of Bak1, Casp3, Casp9	25% dried plum compared to Ovx control: Whole body and vertebra BMA, BMD, and BMC were significantly ↑, vertebra and tibia BV/TV were significantly ↑; vertebra Tb N, Tb Th, Tb Sp, ConnDens and trabecular density were significantly ↑ and SMI was significantly ↓; proximal tibia Tb N and trabecular density were significantly ↑; vertebra total force, stiffness, and size independent stiffness and tibia size independent stiffness were significantly ↑; plasma GPx was significantly ↑; NFATc1 was significantly ↓ and Col1a1 tended to be ↑, bone marrow Bak1 was significantly ↑, and Casp9 was significantly ↓
Smith et al., 2014 [37]	Sham and Ovx female 6-month old Sprague-Dawley rats	84	(1) Sham control, (2) Ovx control, (3) Ovx + 5% dried plum, (4) Ovx + 15% dried plum, (5) Ovx + 25% dried plum, (6) Ovx + PTH	6 weeks	BMA, BMC, and BMD of the whole body, femur, and 4th and 5th lumbar vertebrae; bone microarchitecture parameters of tibia and 4th lumbar vertebrae (BV/TV, Tb N, Tb S, Tb Th, ConnDens, and SMI); dynamic bone histomorphometry (proximal tibia metaphysis BFR, MS/BS, and MAR; tibial cancellous MS/bone area, and BFR/BV; tibial cortical bone periosteal BFR, periosteal MS, periosteal MAR, endocortical BFR, endocortical MS, and endocortical MAR), plasma PINP; urinary Dpd; bone gene expression of NFATc1, Col1a1, ALP, osteocalcin, Runx2, BMP 2 and 4, IFG-1, RANKL, OPG	Compared to Ovx control: Whole body and femur BMD was significantly ↑ in all dried plum groups; vertebral BMD was significantly ↑ in 15% and 25% dried plum groups and tended to be significantly increased in 5% dried plum group; vertebral BV/TV and Tb N significantly ↑ and Tb S and ConnDens significantly ↓ in all dried plum groups, whereas Tb Th and SMI ↑ and ↑, respectively, in 15% and 25% dried plum groups; proximal tibia metaphysis Tb Sp ↓ in 15% and 25% dried plum groups; tibial mid-diaphysis cortical thickness ↑ in all dried plum groups while cortical area tended to be significantly ↑ in 5% dried plum group; plasma PINP was significantly ↓ in all dried plum groups; urinary Dpd tended to be significantly decreased and was significantly decreased in 5% and 15% dried plum groups, respectively; proximal tibial metaphysis BFR tended to be significantly ↓ in 5% dried plum group and was significantly ↓ in 15% and 25% dried plum groups, MS/BS was significantly ↓ in 15% and 25% dried plum groups, and MAR was significantly ↓ in 15% dried plum group and tended to be significantly ↓ in 5% and 25% dried plum groups; tibial cancellous MS/bone area and BFR/BV were significantly ↓ in all dried plum groups; tibial endocortical MAR was significantly ↓ in the 5% dried plum group and tended to be significantly reduced in the 15% and 25% dried plum groups; relative abundance of NFATc1 was significantly ↓ and BMP4 was significantly ↑ in all dried plum groups, while Col1a1 tended to be significantly reduced in all dried plum groups and IGF-1 was significantly ↑ in 25% dried plum group and tended to be significantly ↑ in 15% dried plum group
Pawlowski et al., 2014 [39]	Ovx female 3-month old Sprague-Dawley rats	44	(1) Control, (2) grape seed extract-high, (3) grape seed extract-low, (4) blueberry-high, (5) blueberry-low, (6) dried plum-high, (7) dried plum-low, (8) grape-high, (9) grape-low, (10) resveratrol-high, (11) resveratrol-low, (12) soy isoflavone-glycosylated, (13) soy isoflavone-genistein aglycone	10 days	Urine calcium (^45^Ca and total calcium); serum BALP; urinary NTx	Bone calcium retention was significantly ↑ in dried plum-high group compared to baseline
Léotoing et al., 2016 [40]	Ovx female 5-month old Wistar rats	84	(1) Sham control, (2) Ovx control, (3) high chlorogenic acid dried plum, (4) low chlorogenic acid dried plum, (5) high chlorogenic acid dried plum juice, (6) low chlorogenic acid dried plum juice, (7) low chlorogenic acid dried plum + fiber	90 days	Total femoral BMD, metaphyseal BMD, and diaphyseal BMD; total BMC; blood osteocalcin, CPII, CTX-II, and CRI; urinary Dpd and calcium; BRI	Compared to Ovx control: Total femoral BMD and metaphyseal BMD were significantly ↑ in low chlorogenic acid dried plum + fiber and low chlorogenic acid dried plum juice group and tended to be significantly ↑ in all other dried plum groups; diaphyseal BMD was significantly ↑ in high chlorogenic acid dried plum, low chlorogenic acid dried plum + fiber and the low chlorogenic acid dried plum juice groups; total BMC was significantly ↑ in all low chlorogenic acid dried plum groups and tended to be significantly ↑ in high chlorogenic acid dried plum groups; blood osteocalcin was significantly ↓ in high chlorogenic acid dried plum, low chlorogenic acid dried plum + fiber, and low chlorogenic acid dried plum juice groups and tended to be significantly ↓ in the other dried plum groups; urinary Dpd was significantly ↓ in low chlorogenic acid dried plum + fiber and low chlorogenic acid dried plum juice groups and tended to be significantly ↓ in the other dried plum groups; BRI tended to be ↓ in all high chlorogenic acid dried plum groups and tended to be ↑ in all low chlorogenic acid dried plum groups; all groups had significantly ↑ calciurea; CPII and CRI were significantly ↑ in high chlorogenic acid dried plum group

BALP, bone alkaline phosphatase; BMA, bone mineral area; BMC, bone mineral content; BMD, bone mineral density; BFR, bone formation rate; BMP, bone morphogenetic proteins; BRI, bone remodeling index; BSAP, bone-specific alkaline phosphatase; BV/TV, bone volume to total volume ratio; ^45^Ca, calcium-45; Col1a1, type 1 collagen; ConnDens, connectivity density; CPII, C-propeptide of type II collagen; CRI, cartilage remodeling index; CTX-II, C-terminal telopeptides of type II collagen; Dpd, deoxypyridinoline; HP, helical peptide; IGF-1, insulin-like growth factor-1, IGFBP-3, insulin-like growth factor binding protein-3; IL-6, interleukin-6; MAR, mineral apposition rate; MS, mineralizing surface; MS/BS, mineral surface as percent of bone surface; NFATc1, nuclear factor of activated T cells; NTx, cross-linked N-telopeptides of type 1 collagen; OPG, osteoprotegerin; PINP, N-terminal propeptide of type 1 procollagen; PTH, parathyroid hormone; RANKL, receptor activator of nuclear factor kappa-B ligand; Runx2, runt-related protein 2; Tb N, trabecular number; Tb Sp, trabecular separation; Tb Th, trabecular thickness; TRAP, tartrate-resistant acid phosphatase; TNF-α, tumor necrosis factor-alpha; Ovx, ovariectomized; Sham, sham-operated; SMI, structural model index.

**Table 2:** Clinical trials investigating the role of dried plum on bone health in postmenopausal women.

Reference	Design	Population	Number	Intervention	Duration	Primary Outcomes	Primary Findings
Arjmandi et al., 2002 [41]	RCT	Postmenopausal women	58	100 g/day dried plum or 75 g/day dried apple (comparative control)	3 months	Serum IGF-1, IGFBP-3, AP, TRAP, BSAP, calcium, phosphorus, and magnesium, urinary Dpd, HP, and creatinine	↑ IGF-1, AP, and BSAP compared to baseline in dried plum group
Hooshmand et al., 2011 [13]	RCT	Postmenopausal women with osteopenia	160	100 g/day dried plum or 75 g/day dried apple (comparative control)	12 months	Whole body, lumbar spine, hip, and forearm BMD; serum BALP, osteocalcin, TRAP-5b, and CRP	↑ ulna and lumbar spine BMD in dried plum group compared to dried apple (*p* < 0.05), ↓ BALP in dried plum group compared to baseline
Hooshmand et al., 2014 [44]	RCT	Postmenopausal women with osteopenia	160	100 g/day dried plum or 75 g/day dried apple (comparative control)	12 months	Serum Dpd, RANKL, OPG, and sclerostin	Non-significant ↑ in RANKL, RANKL/OPG ratio, and sclerostin, and ↓ in OPG compared to baseline in dried apple group, non-significant ↑ in OPG and RANKL and ↓ in sclerostin in dried plum group compared to baseline
Simonavice et al., 2014 [45]	Non-randomized intervention trial	Postmenopausal breast cancer survivors	23	Resistance exercise with/without 90 g/day dried plum	6 months	Whole body, lumbar spine, femur, and forearm BMD; serum BAP, TRAP-5b, and CRP	No significant effects
Hooshmand et al., 2016 [46]	RCT	Older postmenopausal women	48	0, 50, or 100 g/day dried plum	6 months	Whole body, lumbar spine, hip, and forearm BMD; serum hs-CRP, IGF-1, BAP, TRAP-5b, BAP/TRAP-5b ratio, sclerostin, 25-OH vitamin D, RANKL, OPG, calcium, and phosphorus	↑ whole body BMD in both dried plum groups compared to control, ↓ TRAP-5b at 3 and 6 months in dried plum groups compared to control, ↑ BAP/TRAP-5b ratio at 6 months in both dried plum groups compared to baseline

BMD, bone mineral density; BSAP, bone-specific alkaline phosphatase; CRP, C-reactive protein; Dpd, deoxypyridinoline; HP, helical peptide; hs-CRP, high-sensitivity CRP; IGF-1, insulin-like growth factor-1, IGFBP-3, insulin-like growth factor binding protein-3; OPG, osteoprotegerin; RANKL, receptor activator of nuclear factor kappa-B ligand; RCT, randomized controlled trial; TRAP, tartrate-resistant acid phosphatase.
